# Supplementary figures and images for: Serotypes, virulence factors and multilocus sequence typing of Glaesserella parasuis from diseased pigs in Taiwan
Source: PeerJ. 2023 Sep 29;11:e15823. doi: 10.7717/peerj.15823 (PMC10544350; doi:10.7717/peerj.15823)

## MLST (&lt;All Characters&gt;)

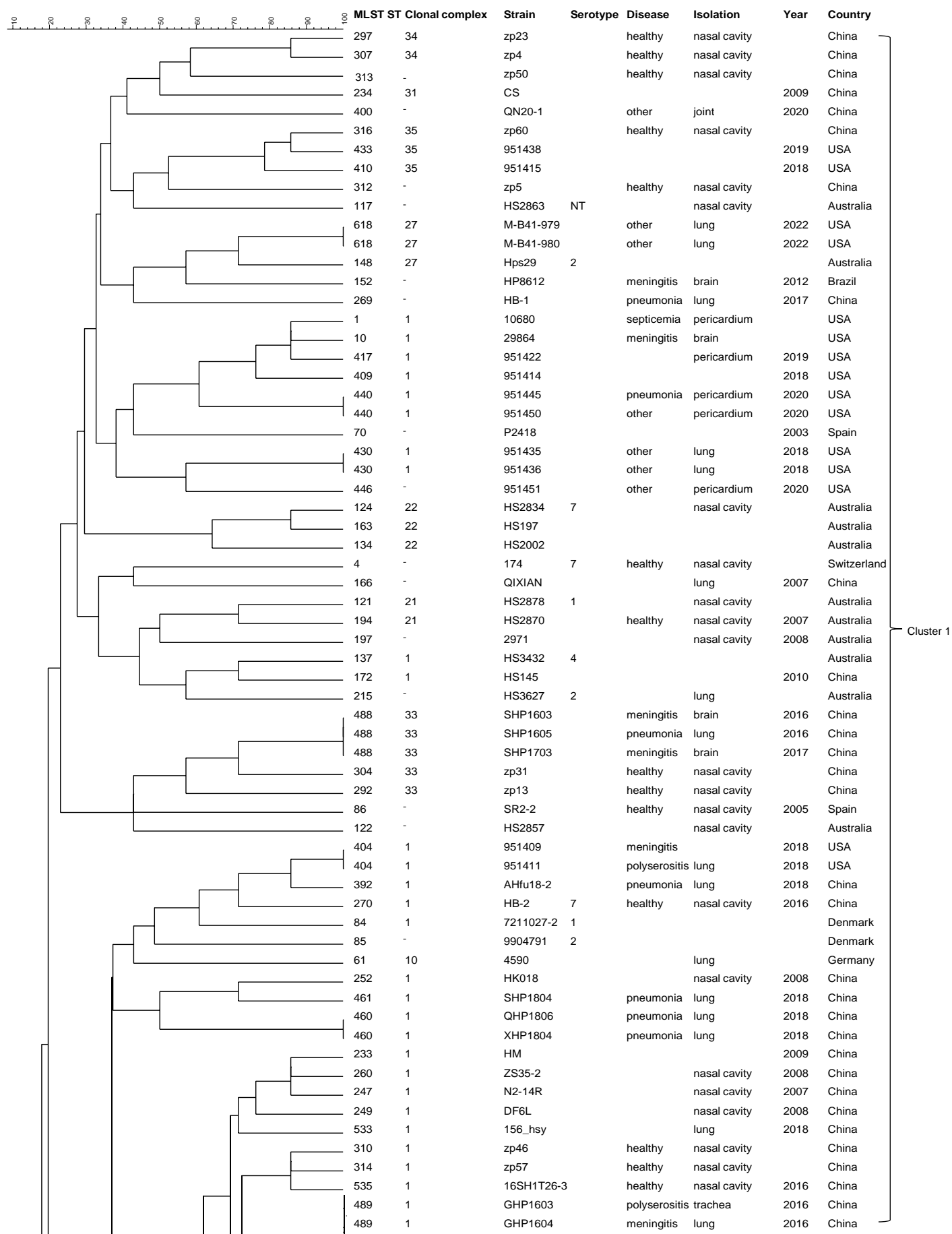

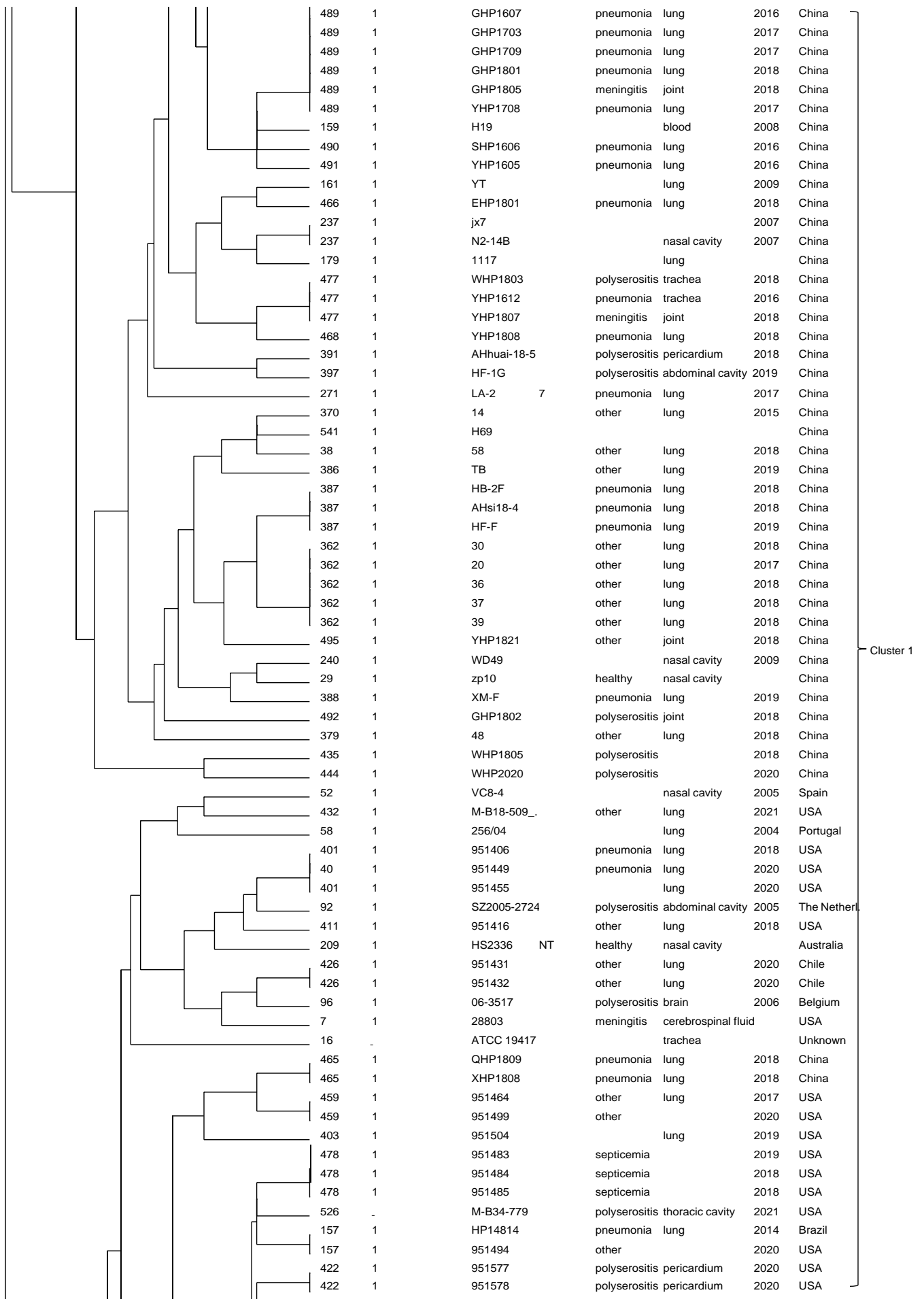

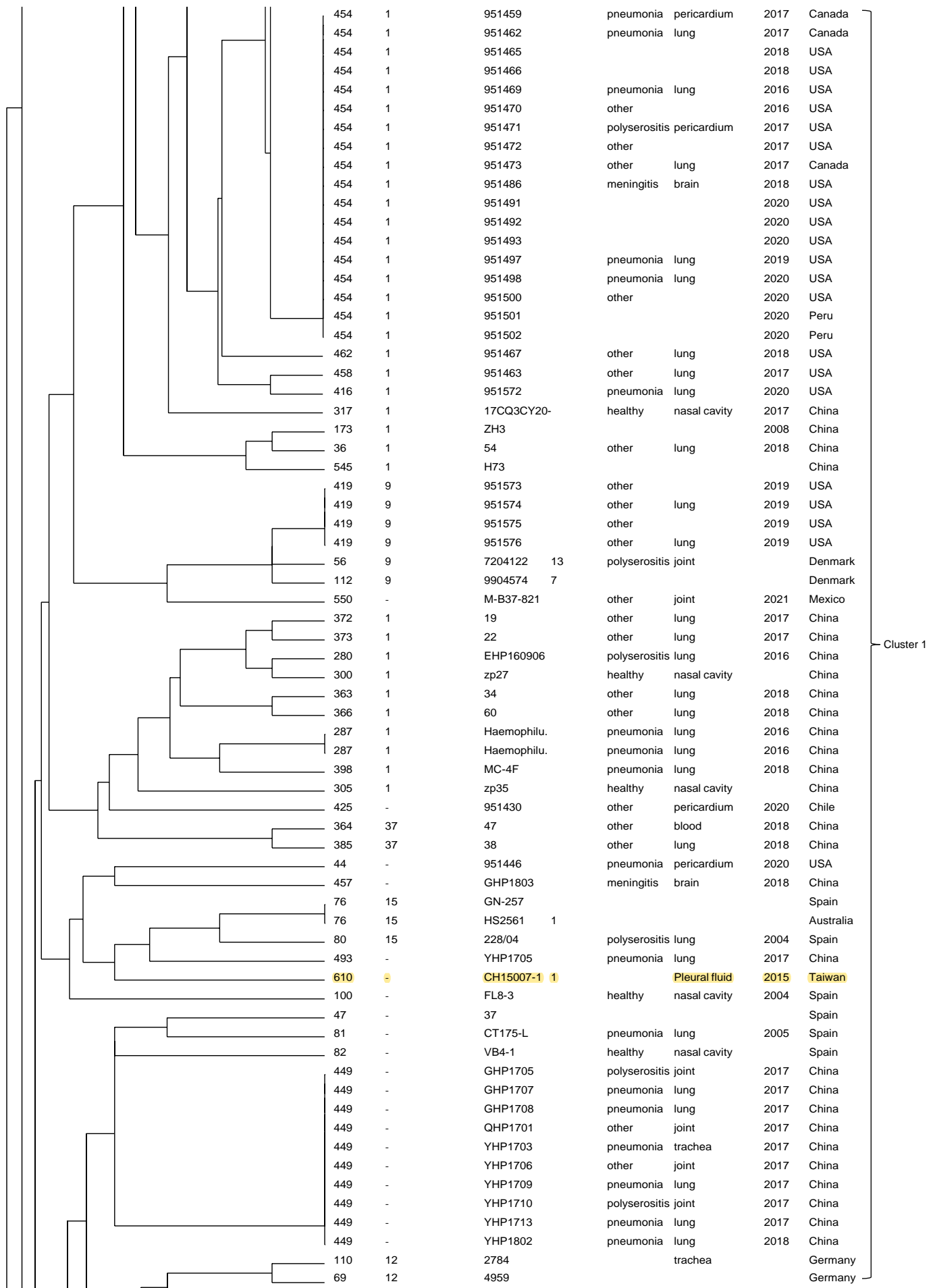

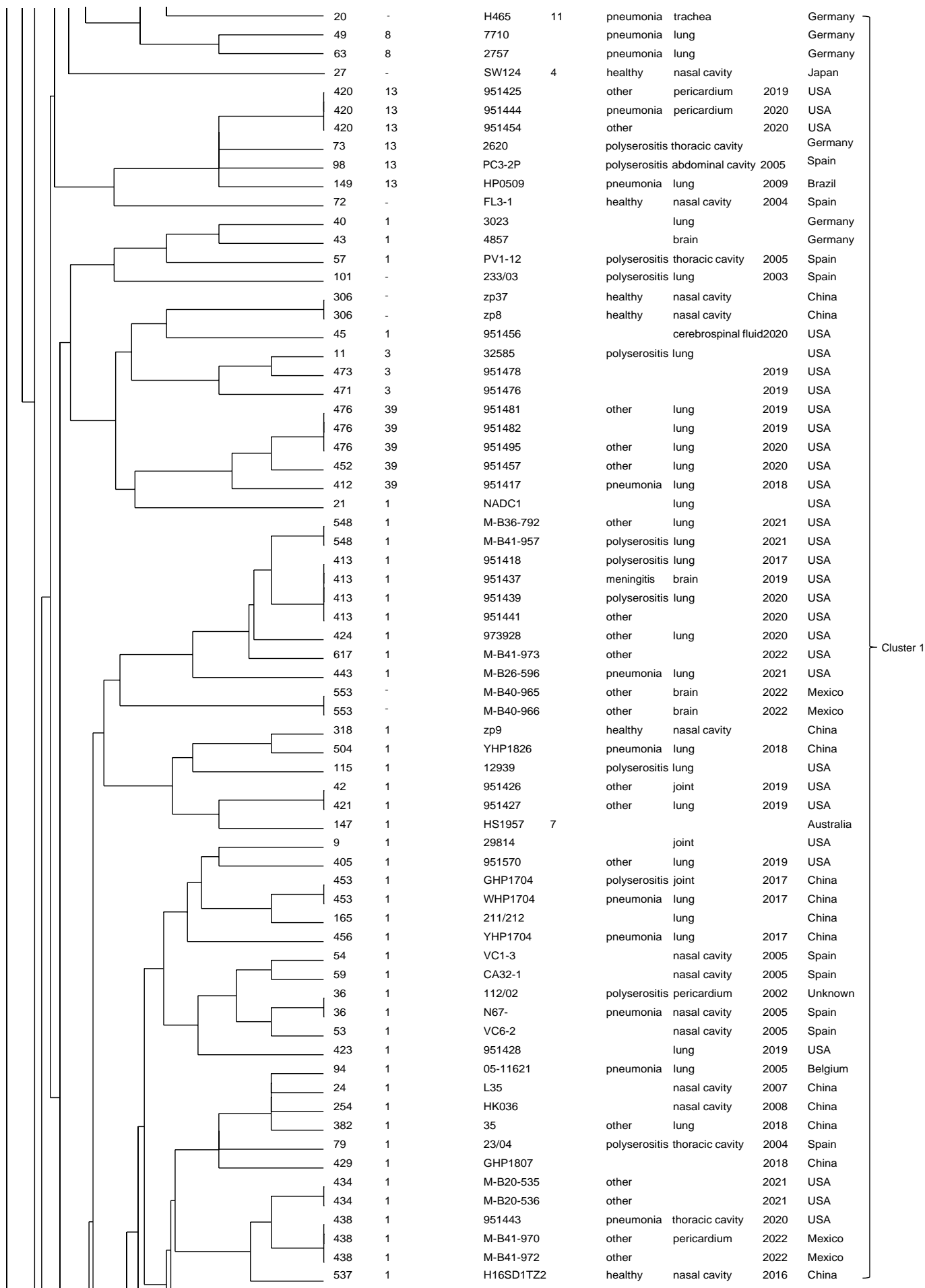

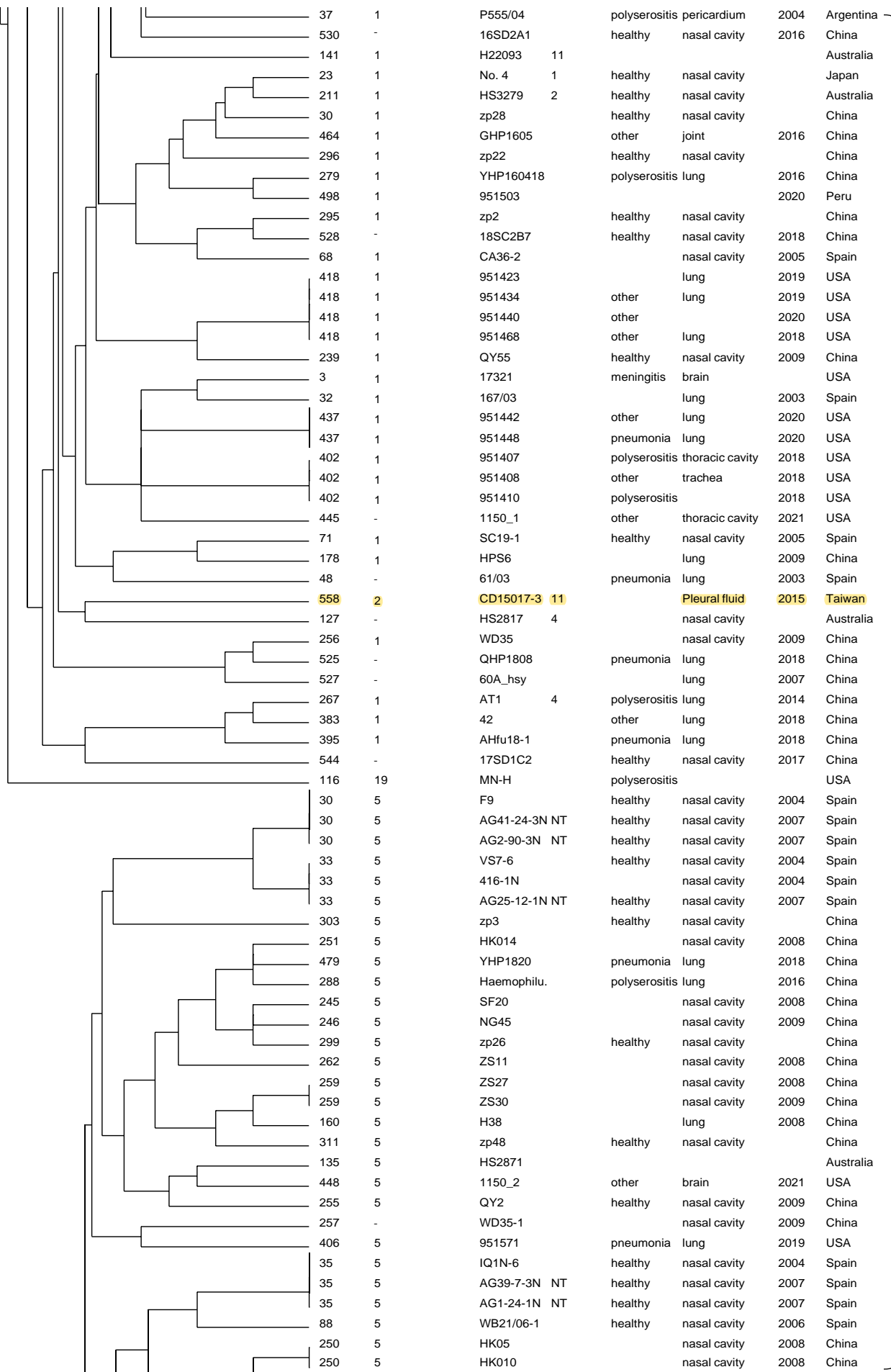

Cluster 1

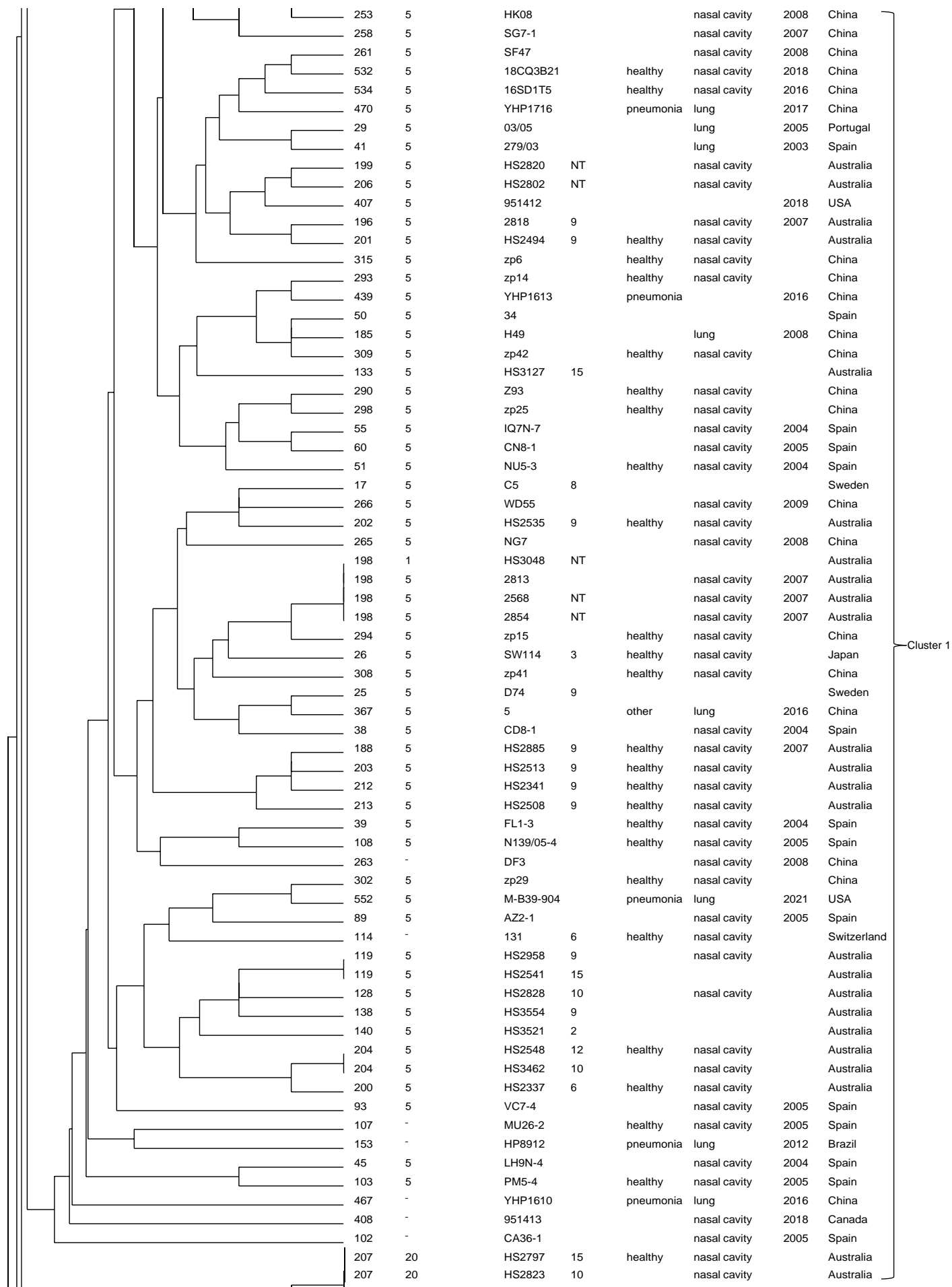

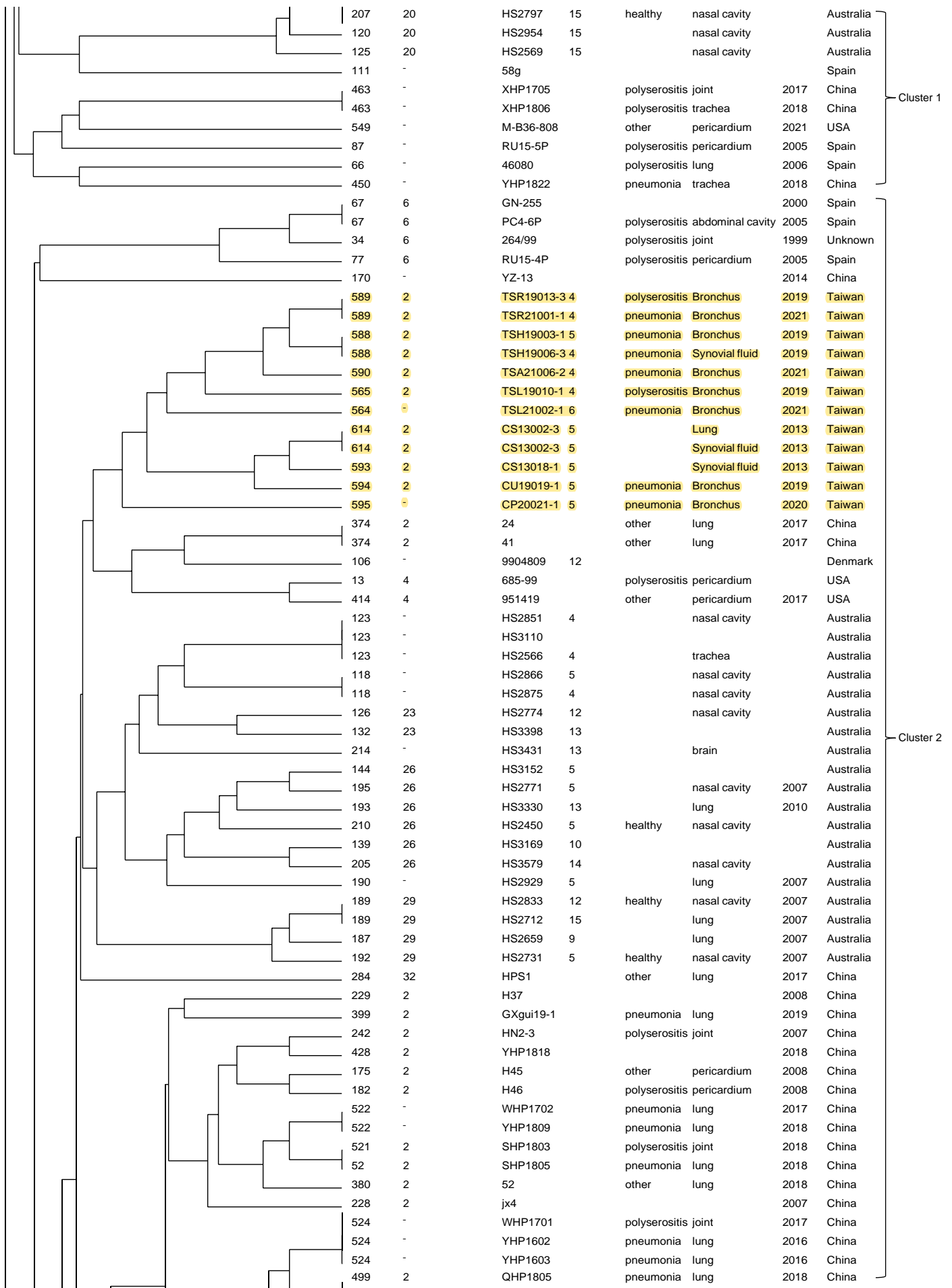

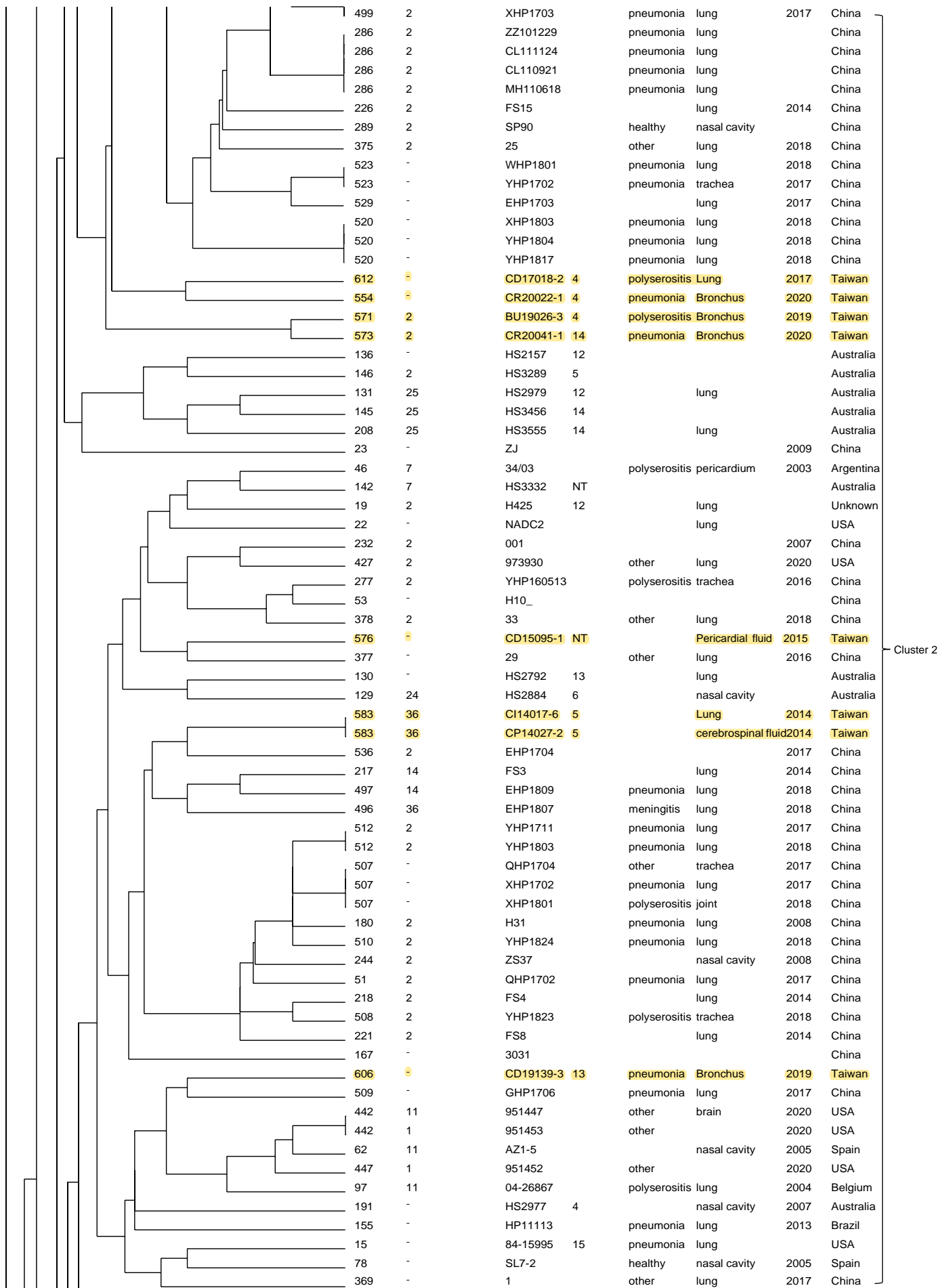

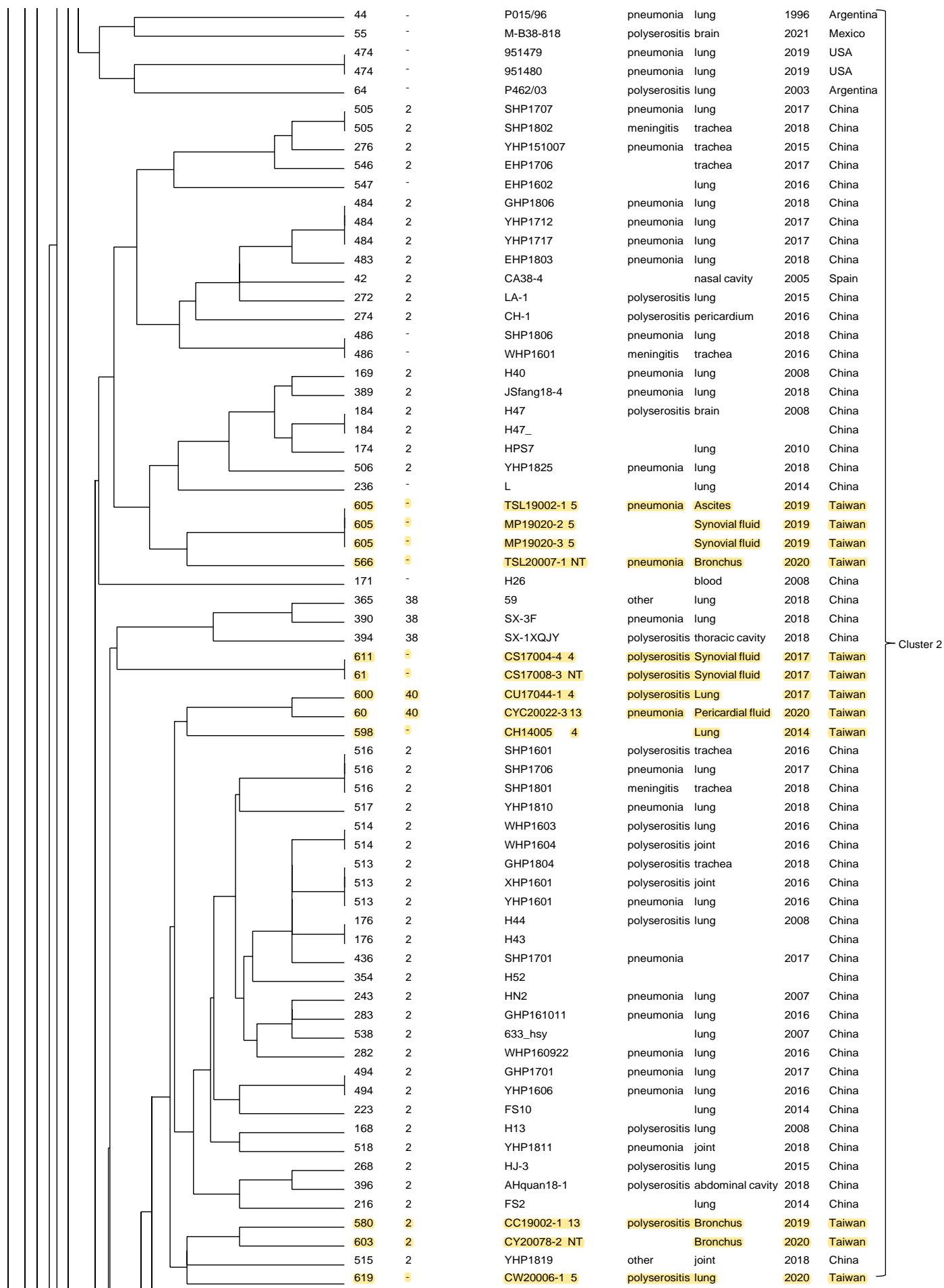

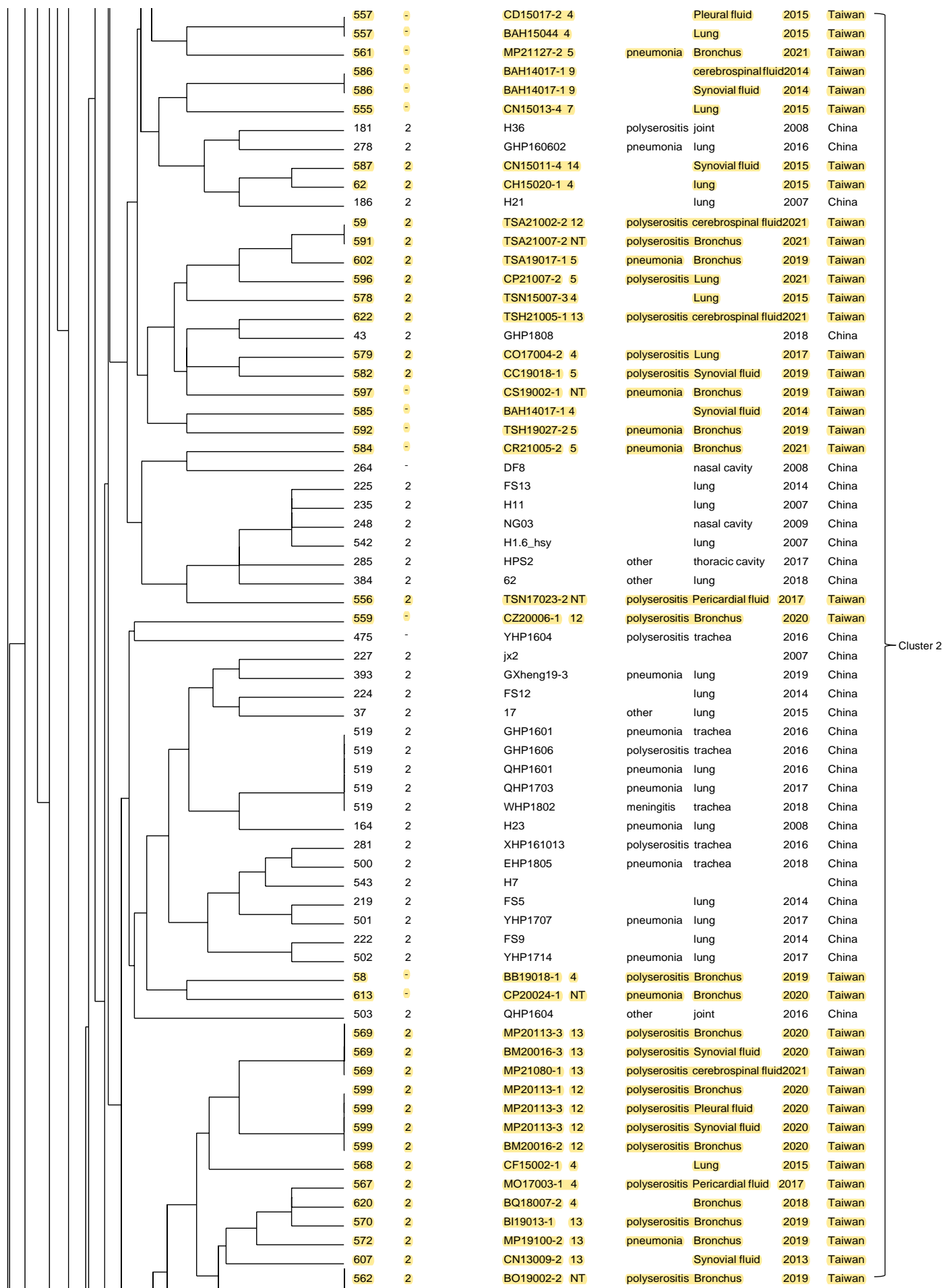

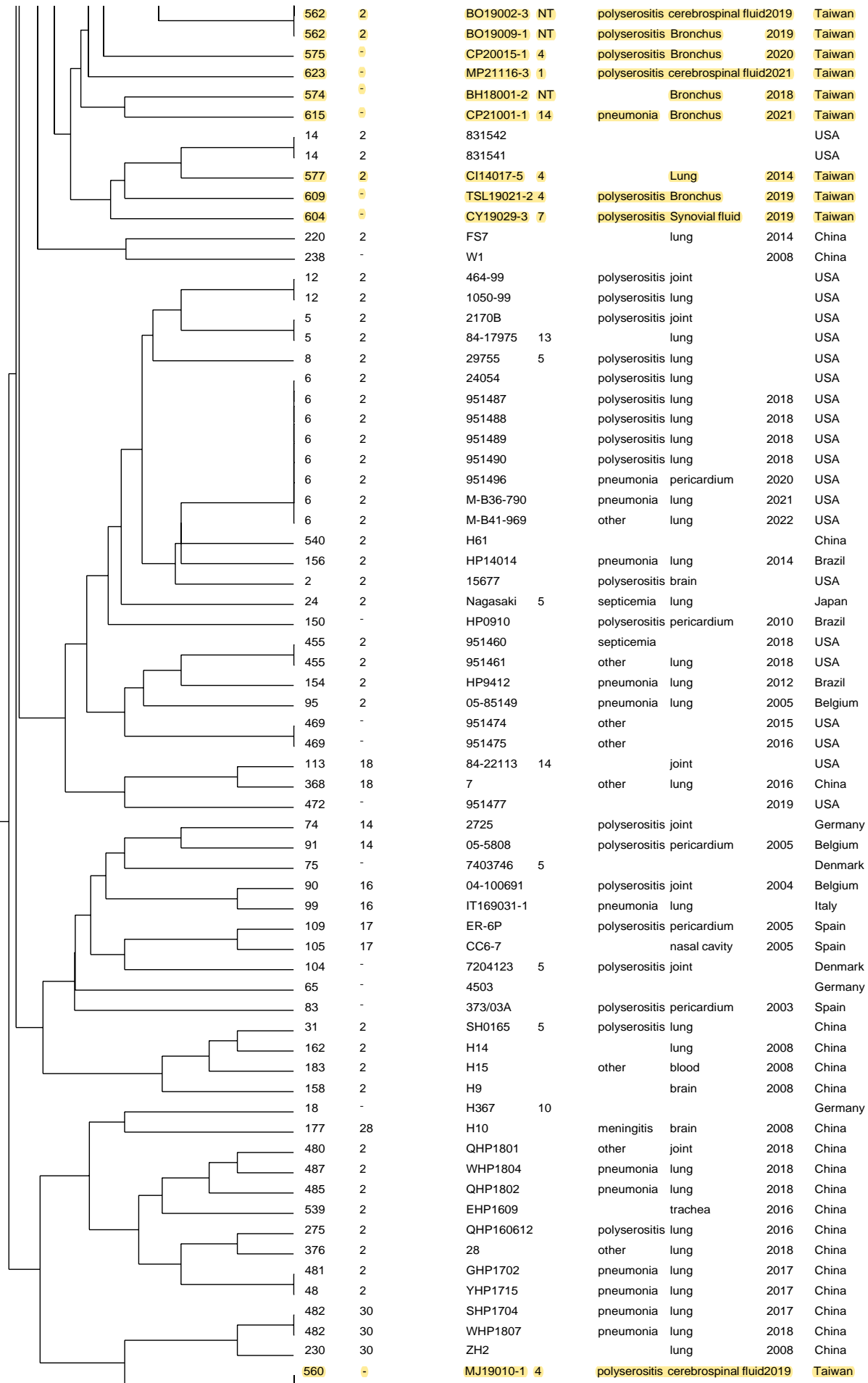

Cluster 2

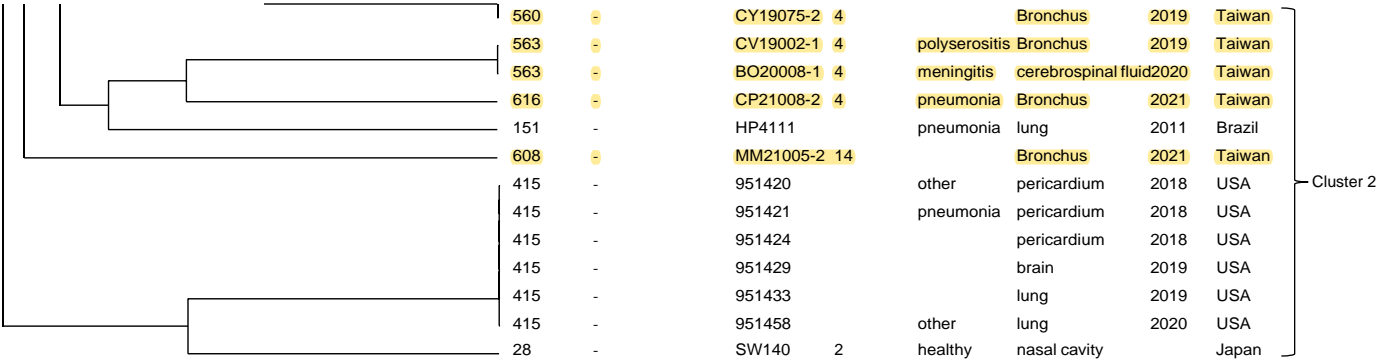

Supplement: Supplemental Information 1 [file peerj-11-15823-s001.pdf]
