# Supplementary material for: Serotypes, virulence factors and multilocus sequence typing of Glaesserella parasuis from diseased pigs in Taiwan
Source: PeerJ. 2023 Sep 29;11:e15823. doi: 10.7717/peerj.15823 (PMC10544350; doi:10.7717/peerj.15823)
Supplement: Supplemental Information 4 [file peerj-11-15823-s004.docx]

**Table S1** Distribution of serotypes per year

|  | Serotype 1 | Serotype 4 | Serotype 5 | Serotype 6 | Serotype 7 | Serotype 9 | Serotype 11 | Serotype 12 | Serotype 13 | Serotype 14 | UT | Total |
| --- | --- | --- | --- | --- | --- | --- | --- | --- | --- | --- | --- | --- |
| 2013 | 0  (0%) | 0  (0%) | 5  (8.4%) | 0  (0%) | 0  (0%) | 0  (0%) | 0  (0%) | 0  (0%) | 1  (3.7%) | 0  (0%) | 0  (0%) | 6 |
| 2014 | 0  (0%) | 9  (9.7%) | 6  (10.2%) | 0  (0%) | 0  (0%) | 1  (25%) | 0  (0%) | 2  (10.5%) | 1  (3.7%) | 0  (0%) | 1  (2.3%) | 20 |
| 2015 | 1  (20%) | 8  (8.6%) | 5  (8.4%) | 0  (0%) | 2  (22.2%) | 0  (0%) | 1  (100%) | 2  (10.5%) | 2  (7.4%) | 1  (6.7%) | 1  (2.3%) | 23 |
| 2016 | 0  (0%) | 0  (0%) | 3  (5.1%) | 0  (0%) | 0  (0%) | 0  (0%) | 0  (0%) | 0  (0%) | 0  (0%) | 0  (0%) | 0  (0%) | 3 |
| 2017 | 0  (0%) | 8  (8.6%) | 6  (10.2%) | 0  (0%) | 0  (0%) | 3  (75%) | 0  (0%) | 1  (5.3%) | 1  (3.7%) | 2  (13.3%) | 6  (13.6%) | 27 |
| 2018 | 2  (40%) | 12  (13%) | 3  (5.1%) | 0  (0%) | 1  (11.1%) | 0  (0%) | 0  (0%) | 1  (5.3%) | 1  (3.7%) | 1  (6.7%) | 13  (29.5%) | 34 |
| 2019 | 1  (20%) | 24 (26.1%) | 15  (25.4%) | 0  (0%) | 4  (44.4%) | 0  (0%) | 0  (0%) | 2  (10.5%) | 9  (33.3%) | 2  (13.3%) | 13  (29.5%) | 70 |
| 2020 | 0  (0%) | 18  (19.6%) | 6  (10.2%) | 0  (0%) | 1  (11.1%) | 0  (0%) | 0  (0%) | 5  (26.3%) | 6  (22.2%) | 3  (20%) | 5  (11.4%) | 44 |
| 2021 | 1  (20%) | 13  (14.1%) | 10  (16.9%) | 1  (100%) | 1  (11.1%) | 0  (0%) | 0  (0%) | 6  (31.6%) | 6  (22.2%) | 6  (40%) | 5  (11.4%) | 49 |
| Total | 5 | 92 | 59 | 1 | 9 | 4 | 1 | 19 | 27 | 15 | 44 | 276 |

**Table S2** Association between *G. Parasuis* serotypes and antimicrobial resistance

| **Serotypes** | Serotype 1  (n = 5) | | Serotype 4  (n = 92) | | Serotype 5  (n = 59) | | Serotype 6  (n = 1) | | Serotype 7  (n = 9) | | Serotype 9  (n = 4) | | Serotype 11  (n = 1) | | Serotype 12  (n = 19) | | Serotype 13  (n = 27) | | Serotype 14  (n = 15) | | ^#^NT  (n = 44) | | Total  (n = 276) | |
| --- | --- | --- | --- | --- | --- | --- | --- | --- | --- | --- | --- | --- | --- | --- | --- | --- | --- | --- | --- | --- | --- | --- | --- | --- |
|  | No. | R% | No. | R% | No. | R% | No. | R% | No. | R% | No. | R% | No. | R% | No. | R% | No. | R% | No. | R% | No. | R% | No. | R% |
| **Antimicrobials** |  |  |  |  |  |  |  |  |  |  |  |  |  |  |  |  |  |  |  |  |  |  |  |  |
| Penicillin | 0 | 0 | 26 | 28.3 | 15 | 25.4 | 1 | 100 | 1 | 11.1 | 3 | 75.0 | 0 | 0 | 5 | 26.3 | 9 | 33.3 | 1 | 6.7 | 11 | 25.0 | 72 | 26.1 |
| Amoxicillin | 3 | 60.0 | 64 | 69.6 | 47 | 79.7 | 1 | 100 | 4 | 44.4 | 4 | 100 | 1 | 100 | 16 | 84.2 | 21 | 77.8 | 7 | 46.7 | 34 | 77.3 | 202 | 73.2 |
| Ceftiofur | 0 | 0 | 3 | 3.3 | 2 | 3.4 | 0 | 0 | 0 | 0 | 0 | 0 | 0 | 0 | 1 | 5.3 | 0 | 0 | 0 | 0 | 0 | 0 | 6 | 2.2 |
| Clarithromycin | 2 | 40.0 | 51 | 55.4 | 43 | 72.9 | 1 | 100 | 2 | 22.2 | 3 | 75.0 | 1 | 100 | 12 | 63.2 | 17 | 63.0 | 7 | 46.7 | 28 | 63.6 | 167 | 60.5 |
| Florfenicol | 0 | 0 | 9 | 9.8 | 3 | 5.1 | 1 | 100 | 0 | 0 | 0 | 0 | 0 | 0 | 0 | 0 | 0 | 0 | 0 | 0 | 3 | 6.8 | 16 | 5.8 |
| Tiamulin | 0 | 0 | 13 | 14.1 | 5 | 8.5 | 0 | 0 | 0 | 0 | 0 | 0 | 0 | 0 | 2 | 10.5 | 3 | 11.1 | 1 | 6.7 | 1 | 2.3 | 25 | 9.1 |
| Trimethoprim/  sulfamethoxazole | 3 | 60.0 | 73 | 79.3 | 53 | 89.8 | 1 | 100 | 7 | 77.8 | 3 | 75.0 | 1 | 100 | 16 | 84.2 | 23 | 85.2 | 11 | 73.3 | 37 | 84.1 | 228 | 82.6 |
| Enrofloxacin | 3 | 60.0 | 61 | 66.3 | 49 | 83.1 | 0 | 0 | 5 | 55.6 | 4 | 100 | 1 | 100 | 16 | 84.2 | 17 | 63.0 | 10 | 66.7 | 29 | 66.0 | 195 | 70.7 |

^#^NT: non-typable

PG: penicillin G, AMO: amoxicillin, CEF: ceftiofur, D: doxycycline, CL: clarithromycin, FFC: florfenicol, TIA: tiamulin, ENR: enrofloxacin, TS: trimethopri-sulfamethoxazole.

**Table S3** Association between isolation sites of *G. Parasuis* and antimicrobial resistance

| **Isolation sites** | Lower respiratory tract  (n = 188) | | Synovial fluid  (n = 49) | | Coelomic fluid  (n = 30) | | Cerebrospinal fluid  ( n = 9) | | Total  (n = 276) | |
| --- | --- | --- | --- | --- | --- | --- | --- | --- | --- | --- |
|  | No. | R% | No. | R% | No. | R% | No. | R% | No. | R% |
| **Antimicrobials** |  |  |  |  |  |  |  |  |  |  |
| Penicillin | 47 | 25.0 | 17 | 34.7 | 6 | 20.0 | 2 | 22.2 | 72 | 26.1 |
| Amoxicillin | 135 | 71.8 | 36 | 73.5 | 24 | 80.0 | 7 | 77.8 | 202 | 73.2 |
| Ceftiofur | 5 | 2.7 | 0 | 0 | 1 | 3.3 | 0 | 0 | 6 | 2.2 |
| Clarithromycin | 111 | 59.0 | 32 | 65.3 | 18 | 60.0 | 6 | 66.7 | 167 | 60.5 |
| Florfenicol | 10 | 5.3 | 4 | 8.2 | 0 | 0 | 2 | 22.2 | 16 | 5.8 |
| Tiamulin | 14 | 7.4 | 7 | 14.3 | 3 | 10.0 | 1 | 11.1 | 25 | 9.1 |
| Trimethoprim/  sulfamethoxazole | 154 | 81.9 | 41 | 83.7 | 25 | 83.3 | 8 | 88.9 | 228 | 82.6 |
| Enrofloxacin | 126 | 67.0 | 39 | 79.6 | 22 | 73.3 | 8 | 88.9 | 195 | 70.7 |

**Table S4** Microorganisms co-infected with *G. Parasuis*

| Additional microorganisms in the samples | | Co-infection with *G. Parasuis* | | Number of samples tested |
| --- | --- | --- | --- | --- |
|  |  | N | % |  |
| Type | Pathogen |  |  |  |
| Virus | Porcine reproductive and respiratory syndrome virus (PRRSV) | 232 | 84.1 | 276 |
|  | Porcine circovirus type 2 (PCV2) | 33 | 12.0 | 276 |
|  | Porcine cytomegalovirus (PCMV) | 14 | 5.1 | 276 |
| Bacteria | *Mycoplasma hyorhinis* | 111 | 40.2 | 276 |
|  | *Mycoplasma hyosynoviae* | 7 | 2.5 | 276 |
|  | *Pasteurella multocida* | 20 | 7.2 | 276 |
|  | *Streptococcus suis* | 58 | 21.0 | 276 |
|  | *Mycoplasma suis* | 63 | 22.8 | 276 |

**Table S5** Association between *G. Parasuis* serotypes and virulence genes

| **Serotypes** | Serotype 1  (n = 5) | | Serotype 4  (n = 92) | | Serotype 5  (n = 59) | | Serotype 6  (n = 1) | | Serotype 7  (n = 9) | | Serotype 9  (n = 4) | | Serotype 11  (n = 1) | | Serotype 12  (n = 19) | | Serotype 13  (n = 27) | | Serotype 14  (n = 15) | | NT  (n = 44) | | Total  (n = 276) | |
| --- | --- | --- | --- | --- | --- | --- | --- | --- | --- | --- | --- | --- | --- | --- | --- | --- | --- | --- | --- | --- | --- | --- | --- | --- |
|  | No. | R% | No. | R% | No. | R% | No. | R% | No. | R% | No. | R% | No. | R% | No. | R% | No. | R% | No. | R% | No. | R% | No. | R% |
| **Virulence gene** |  |  |  |  |  |  |  |  |  |  |  |  |  |  |  |  |  |  |  |  |  |  |  |  |
| *vtaA* | 5 | 100 | 92 | 100 | 59 | 100 | 1 | 100 | 9 | 100 | 4 | 100 | 1 | 100 | 19 | 100 | 27 | 100 | 15 | 100 | 44 | 100 | 276 | 100 |
| *fhuA* | 4 | 80.0 | 71 | 77.2 | 53 | 89.8 | 0 | 0 | 9 | 100 | 4 | 100 | 1 | 100 | 11 | 57.9 | 23 | 85.2 | 13 | 86.7 | 33 | 75.0 | 222 | 80.4 |
| *hhdA* | 4 | 80.0 | 90 | 97.8 | 59 | 100 | 1 | 100 | 9 | 100 | 4 | 100 | 1 | 100 | 19 | 100 | 26 | 96.3 | 15 | 100 | 44 | 100 | 272 | 98.6 |
| *hhdB* | 4 | 80.0 | 90 | 97.8 | 55 | 93.2 | 1 | 100 | 9 | 100 | 4 | 100 | 1 | 100 | 19 | 100 | 26 | 96.3 | 14 | 93.3 | 42 | 95.5 | 265 | 96.0 |
| *nhaC* | 4 | 80.0 | 91 | 98.9 | 58 | 98.3 | 1 | 100 | 9 | 100 | 4 | 100 | 1 | 100 | 19 | 100 | 25 | 92.6 | 15 | 100 | 44 | 100 | 271 | 98.2 |
| *HAPS_0254* | 4 | 80.0 | 87 | 94.6 | 48 | 81.4 | 1 | 100 | 8 | 88.9 | 4 | 100 | 1 | 100 | 16 | 84.2 | 19 | 70.4 | 13 | 86.7 | 36 | 81.8 | 237 | 85.9 |
| *cirA* | 4 | 80.0 | 92 | 100 | 59 | 100 | 1 | 100 | 9 | 100 | 4 | 100 | 1 | 100 | 19 | 100 | 26 | 96.3 | 15 | 100 | 44 | 100 | 274 | 99.3 |
| *sclB7* | 5 | 100 | 92 | 100 | 59 | 100 | 1 | 100 | 9 | 100 | 4 | 100 | 1 | 100 | 19 | 100 | 27 | 100 | 14 | 93.3 | 44 | 100 | 275 | 99.6 |
| *sclB11* | 5 | 100 | 89 | 96.7 | 57 | 96.6 | 1 | 100 | 9 | 100 | 4 | 100 | 1 | 100 | 17 | 89.5 | 26 | 96.3 | 11 | 73.3 | 42 | 95.5 | 262 | 94.9 |
| *phage_related* | 2 | 40 | 11 | 12.0 | 2 | 3.4 | 0 | 0 | 0 | 0 | 1 | 25.0 | 0 | 0 | 0 | 0 | 1 | 3.7 | 4 | 26.7 | 2 | 4.5 | 23 | 8.3 |
